# Comparison of endoscopic resection, laparoscopic resection, and laparoscopic endoscopic cooperative surgery in esophageal or gastric subepithelial lesions in a Thai medical school

**DOI:** 10.2478/abm-2025-0026

**Published:** 2025-09-08

**Authors:** Phuphat Vongwattanakit, Voranaddha Vacharathit, Kasaya Tantiphlachiva, Mawin Vongsaisuwon, Chadin Tharavej, Sopark Manasnayakorn, Pornpol Palanusorn

**Affiliations:** Department of Surgery, Faculty of Medicine, Chulalongkorn University, Bangkok 10330, Thailand

**Keywords:** endoscopic resection, esophageal subepithelial lesions, gastric subepithelial lesions, laparoscopic resection, laparoscopic-endoscopic cooperative surgery

## Abstract

**Background:**

Esophageal and gastric subepithelial lesions (SELs) are increasingly identified in routine endoscopic evaluations, necessitating optimal resection strategies. Minimally invasive techniques, including endoscopic resection (ER), laparoscopic resection (LR), and laparoscopic-endoscopic cooperative surgery (LECS), have distinct advantages. The present study compares the outcomes of these techniques.

**Objective:**

To compare the clinical outcomes of ER, LR, and LECS in the management of esophageal or gastric SELs at King Chulalongkorn Memorial Hospital.

**Methods:**

A retrospective review was conducted on patients undergoing ER, LR, or LECS for esophageal and gastric SELs from January 2012 to August 2022. The primary outcome was the complete resection rate. Secondary outcomes included success rates, complications, and length of hospital stay. Statistical significance was set at *P* < 0.05.

**Results:**

Among 42 patients, 11 (26.2%) underwent ER, 12 (28.6%) underwent LR, and 19 (45.2%) underwent LECS. Complete resection was significantly higher in LR (100%) and LECS (84.2%) than ER (45.5%) (*P* = 0.033). Delayed bleeding occurred in 18.2% of ER cases (*P* = 0.052). Hospital stay was shortest in ER (3.9 d) and longest in LR (9.3 d) (*P* = 0.877).

**Conclusion:**

While all techniques had high success rates, ER had the lowest complete resection rate and the highest bleeding risk. LR ensured complete resection but required longer hospitalization. LECS provided a balance between oncologic efficacy and safety. Surgical planning should be tailored based on tumor characteristics and surgical expertise.

Esophageal and gastric subepithelial lesions (SELs) are lesions that originate from the submucosal or muscularis propria layers of the stomach wall and are often detected incidentally during endoscopic or radiologic evaluations. Studies have reported that SELs are found in approximately 0.36%–3% of upper gastrointestinal endoscopies, with the stomach as the most common site [1,2,3]. These tumors include a variety of histological entities, including gastrointestinal stromal tumors (GISTs), leiomyomas, schwannomas, neuroendocrine neoplasm (NENs), and other rare neoplasms. Although many esophageal and gastric SELs are benign and asymptomatic, some exhibit malignant potential, necessitating careful evaluation and appropriate management.

The primary surgical objective in treating patients with esophageal and gastric SELs, particularly GISTs, is to achieve complete tumor resection while minimizing the risk of tumor rupture. Special caution must be taken to prevent tumor spillage or capsular disruption, as such occurrences may lead to peritoneal contamination, increasing the risk of tumor dissemination and recurrence. Notably, lymphadenectomy is not required in the surgical management of these lesions.

Various surgical techniques are available for treating esophageal and gastric SELs, including open surgery, laparoscopic resection (LR), endoscopic resection (ER), and laparoscopic-endoscopic cooperative surgery (LECS), each with its advantages and limitations. The evolution of minimally invasive approaches has been driven by the goal of reducing postoperative morbidity and expediting recovery.

LR is a minimally invasive laparoscopic surgery that involves small incisions, reducing postoperative pain, expediting recovery, and decreasing postoperative complications compared with open surgery. However, the technique is limited in cases where tumors are in difficult-to-access locations, such as the esophagogastric junction (EGJ) or pyloric region, where an aggressive wedge resection may lead to gastric deformity and stricture.

ER has many techniques, such as endoscopic submucosal dissection (ESD), submucosal tunneling endoscopic resection (STER), endoscopic submucosal muscularis resection (ESMR), and endoscopic full-thickness resection (EFTR), that have been developed for the resection of gastric SELs. ESD enables en bloc resection but carries a high risk of perforation, particularly for deeply seated tumors [4]. STER involves creating a submucosal tunnel to access and resect the lesion, followed by mucosal closure. EFTR allows for complete resection of all layers of the gastric wall. However, it poses challenges such as pneumoperitoneum due to gastric perforation, difficulty in maneuvering the endoscope, and the risk of abdominal compartment syndrome. The over-the-scope clip (OTSC) full-thickness resection device (FTRD) is an advanced endoscopic system designed for full-thickness resection of gastrointestinal lesions. The system integrates a preloaded clip and a snare for resection, allowing for en bloc excision of lesions while simultaneously sealing the defect. This technique ensures en bloc resection with minimal risk of peritoneal contamination. Despite its advantages, the OTSC FTRD has several limitations. The device is most effective for lesions ≤2 cm in diameter [5, 6], making it less suitable for larger tumors that may require alternative resection techniques. Additionally, its application is challenging in anatomically difficult locations, such as the gastric fundus and duodenum, where maneuverability is limited. Furthermore, resections performed at the EGJ or pylorus carry a heightened risk of luminal stenosis, potentially leading to postoperative obstruction and requiring further intervention.

Due to the limitations of LR and ER, LECS has emerged as a technique that combines the advantages of endoscopic intraluminal visualization with laparoscopic external access. This approach allows for precise tumor resection with adequate margins while minimizing unnecessary gastric wall excision [7]. Additionally, gastric wall defects can be closed using laparoscopic sutures or staplers, reducing the risk of postoperative stricture, particularly in critical regions such as the EGJ and pylorus. LECS may also reduce the likelihood of gastric stasis when tumors are located along the lesser curvature. Several modifications of LECS, such as inverted LECS, laparoscopy-assisted endoscopic full-thickness resection (LAEFR), the combination of laparoscopic and endoscopic approaches to neoplasia with a non-exposure technique (CLEAN-NET), non-exposed endoscopic wall-inversion surgery (NEWS), and closed LECS, have been developed to further refine the technique and reduce the risk of tumor spillage. The surgical techniques have been previously described in other published studies [8].

Despite the growing adoption of these techniques, comparative studies evaluating their clinical outcomes remain limited, particularly in the context of real-world practice. Understanding the efficacy, safety, and feasibility of these approaches in a tertiary referral hospital setting is crucial for guiding surgical decision-making. The present study aims to compare the outcomes of ER, LR, and LECS in the management of esophageal or gastric SELs at King Chulalongkorn Memorial Hospital, a tertiary care university hospital in Thailand, focusing on factors such as operative success rates, complete resection rates, complication rates, and hospital length of stay.

The primary objective of the present study is to compare the clinical outcomes of ER, LR, and LECS in the management of esophageal or gastric SELs at King Chulalongkorn Memorial Hospital.

Specifically, the present study aims to evaluate and compare the operative success and complete resection rates of ER, LR, and LECS. Additionally, perioperative outcomes— including operative time, intraoperative complications—and postoperative outcomes—such as length of hospital stay and postoperative complications—will be assessed.

## Methods

The present study was approved by the Institutional Review Board (IRB) of King Chulalongkorn Memorial Hospital (COA No. 1102/2024). The requirement for informed consent was waived due to the retrospective design of the study. All data were anonymized to protect patient confidentiality and ensure compliance with ethical standards in biomedical research.

### Patients

The present study is a retrospective analysis of patients diagnosed with esophageal or gastric SELs who underwent surgical resection at King Chulalongkorn Memorial Hospital between January 2012 and August 2022. Patient data were retrieved from medical records, including demographic information, tumor characteristics, surgical procedures, and postoperative outcomes. Patients with a confirmed diagnosis of esophageal or gastric SEL based on preoperative imaging and/or endoscopic findings and who subsequently underwent ER, LR, or LECS for tumor removal were included in the study.

Patients were excluded if they presented with an emergency condition requiring urgent intervention (such as active bleeding, perforation, and gastric outlet obstruction), underwent multi-visceral organ resection or other surgical techniques outside the three studied approaches, or had evidence of metastatic disease. Patients with incomplete medical records, including surgical details and follow-up data, were also excluded.

### Data collection

Patient data were retrospectively retrieved from the electronic medical records and categorized into demographic, operative, and postoperative variables, as described below:

Demographic and clinical data include patient characteristics, tumor characteristics (tumor size, site, type, and location), and patient medical history (use of antiplatelet or anticoagulant therapy).

Operative data include surgical approach (ER, LR, and LECS) and intraoperative parameters (operative time [min], estimated blood loss [mL], conversion rate [proportion of cases requiring conversion to an alternative surgical technique], and intraoperative perforation).

Postoperative data include complications (delayed bleeding, leakage, surgical site infection [SSI], and other postoperative complications), histopathological outcomes (margin status [negative or positive resection margins]), and length of hospital stay (d).

### Outcomes

The primary outcome of the study was the complete resection rate for each surgical technique (ER, LR, and LECS), defined as tumor removal with negative margins on histopathological examination.

The secondary outcomes included the surgical success rate, defined as the successful removal of the lesion using the designated technique without conversion to a different technique, and complication rates, which include intraoperative bleeding, perforation, and postoperative morbidity.

### Statistical analysis

The present study was conducted as a retrospective comparative analysis to evaluate the outcomes of different surgical approaches for esophageal or gastric SELs. Categorical variables were presented as counts and percentages and analyzed using Fisher’s exact test for comparisons between groups. Continuous variables were expressed as mean ± standard deviation (SD) and compared using the independent *t*-test. Statistical significance was defined as *P* < 0.05. All statistical analyses were performed using SPSS version 28 (IBM Corp.).

## Results

### Patients

A total of 110 patients who underwent surgical management for esophageal or gastric SELs at King Chulalongkorn Memorial Hospital were initially included in the present study. After applying the exclusion criteria, 42 patients remained for analysis. Among the 42 patients, 11 (26.2%) underwent ER, 12 (28.6%) underwent LR, and 19 (45.2%) underwent LECS. They comprised 19 males (45.2%) and 23 females (54.8%). The mean age of the study population was 61.8 years (range: 35–85 years). The mean tumor size across all groups was 2.8 cm (range: 0.7–9 cm). The difference in tumor size among groups was not statistically significant (*P* = 0.214). Among the 42 patients, 4 (9.5%) were on antiplatelet therapy, 1 (2.4%) was on anticoagulant therapy, and 37 (88.1%) had no history of antithrombotic medication use (**Table 1**).

**Table 1.** Demographic data of the study population

**Variable**	**N (%)/Mean**	**Min–max**	***P***
Sex			
Male	19 (45.2)	–	–
Female	23 (54.8)	–	–
Age: mean age (years)	61.8	35–85	–
Tumor size (cm)			
Overall	2.8	0.7–9	0.214
Endoscopic	2.7	0.7–4.8	–
Laparoscopic	3.2	1.7–9	–
Combined	2.8	1.3–5.1	–
Antiplatelet/anticoagulant usage			
Antiplatelet use	4 (9.5)	–	–
Anticoagulant use	1 (2.4)	–	–
None	37 (88.1)	–	–

### Tumor location

Esophageal or gastric SELs were distributed throughout the upper gastrointestinal tract, with the majority located on the lesser curvature (n = 9) and antrum (n = 9), followed by the fundus (n = 8). Other locations included the greater curvature (n = 7), EGJ (n = 6), esophagus (n = 3), and gastric body (n = 1).

### Tumor growth pattern

Of the 42 tumors, 9 (21.4%) exhibited an exophytic growth pattern, while 33 (78.6%) were classified as endophytic tumors.

### Histopathological diagnosis

The most common histopathological diagnosis was GIST, accounting for 21 cases (50%), followed by leiomyoma (n = 9, 21.4%) and ectopic pancreas (n = 6, 14.3%). Other less common diagnoses included schwannoma (n = 3, 7.1%), NENs (n = 1, 2.4%), accessory spleen (n = 1, 2.4%), and inflammatory fibroid polyp (n = 1, 2.4%).

### Operative techniques

A total of 42 patients underwent surgical management for esophageal and gastric SELs using three different approaches: ER, LR, and LECS as shown in **Table 2**.

**Table 2.** Operative techniques used in gastric SEL resection

**Surgical approach**	**N (%)**
ER	
Total	11 (26.2)
ESD	5
Hybrid ESD	1
STER	2
ESMR	1
OTSC with FTRD	1
EFTR	1
LR	
Total	12 (28.6)
Wedge resection	8
Anatomical resection	4
LECS	
Total	19 (45.2)
LECS	16
NEWS	1
CLEAN–NET	2

CLEAN–NET, combination of laparoscopic and endoscopic approaches to neoplasia with a non-exposure technique; EFTR, endoscopic full–thickness resection; ER, endoscopic resection; ESD, endoscopic submucosal dissection; ESMR, endoscopic submucosal muscularis resection; FTRD, full-thickness resection device; LECS, laparoscopic– endoscopic cooperative surgery; LR, laparoscopic resection; NEWS, non-exposed endoscopic wall-inversion surgery; OTSC, over-the-scope clip; SEL, subepithelial lesion; STER, submucosal tunneling endoscopic resection.

## Surgical approach based on tumor growth pattern

ER was predominantly used for endophytic tumors (91%), with only one case involving an exophytic tumor (9%). LR was performed on a nearly equal proportion of endophytic (58.3%) and exophytic (41.7%) tumors. LECS was primarily performed for endophytic tumors (84.2%), with a smaller proportion of exophytic tumors (15.8%).

The distribution of surgical approaches based on tumor growth patterns (endophytic vs. exophytic) is summarized in **Table 3**.

**Table 3.** Surgical approach based on tumor growth pattern

**Tumor growth pattern**	**N (%)**
ER	
Total	11 (26.2)
Endophytic	10 (91.0)
Exophytic	1 (9.0)
LR	
Total	12 (28.6)
Endophytic	7 (58.3)
Exophytic	5 (41.7)
LECS	
Total	19 (45.2)
Endophytic	16 (84.2)
Exophytic	3 (15.8)

ER, endoscopic resection; LECS, laparoscopic–endoscopic cooperative surgery; LR, laparoscopic resection.

These findings suggest that there is a surgeon preference for ER and LECS approaches for endophytic tumors, while LR is more commonly used for exophytic tumors.

## Surgical approach based on tumor location

Esophageal tumors were almost treated with ER, reflecting the minimally invasive approach preferred in this area. Tumors at the EGJ were more frequently treated with combined surgery. Fundus, lesser curvature, and antrum tumors were more evenly distributed across all three approaches but slightly favored laparoscopic surgery, indicating that all approaches were considered viable options in these locations.

Tumor location appears to be an important factor in determining the surgical approach selected by the surgeon (**Table 4**).

**Table 4.** Tumor location by surgical approach

**Tumor location**	**ER**	**LR**	**LECS**
Esophagus	3	–	–
EGJ	–	–	6
Fundus	2	4	2
Greater curvature	2	–	5
Lesser curvature	1	4	4
Antrum	3	3	2
Body	–	1	–

EGJ, esophagogastric junction; ER; endoscopic resection; LECS; laparoscopic-endoscopic cooperative surgery; LR; laparoscopic resection.

## Surgical outcomes

### Success rate and complete resection

The overall success rate for all surgical approaches was 95.2%, with no significant difference among groups (*P* = 0.438). ER (90.9%) and LR (91.7%) showed comparable success rates, whereas LECS achieved 100% success. This suggests that all three approaches are highly effective in achieving surgical objectives.

The complete resection rate was significantly different among the groups (*P* = 0.033). LR achieved the highest complete resection rate (100%), followed by LECS (84.2%), while ER had the lowest complete resection rate (45.5%). This indicates that LR and LECS provide more definitive tumor removal, whereas endoscopic techniques may be limited in achieving negative resection margins.

### Operative time

The mean operative time varied among the three approaches, with LR achieving the shortest duration (2.8 h), followed by ER (3.6 h), while LECS required the longest operative time (5.1 h). The difference in operative time between groups was not statistically significant (*P* = 0.880). The longer operative time in LECS reflects the need for synchronized laparoscopic and endoscopic coordination, which may contribute to procedural complexity.

### Complications

ER was associated with an intraoperative perforation rate of 45.5%; however, all cases were successfully managed with endoscopic closure techniques, with no instances of postoperative leakage. This finding highlights the inherent risk of deep-layer injury in endoscopic procedures, particularly in tumors originating from or infiltrating the muscularis propria.

Delayed bleeding was more common in the ER group (18.2%), but this difference was not statistically significant (*P* = 0.052). This may be attributed to the difficulty in achieving hemostasis during endoscopic procedures compared to laparoscopic surgery.

SSI occurred in 8.3% of the LR group and 5.3% of the LECS group.

Other complications, including pulmonary complications and intra-abdominal collections, were observed in 12.7% of the LR group, 5.3% of the LECS group, and none in the ER group (*P* = 0.289). These complications may be related to the complexity of the surgical approach and postoperative pain from surgical wounds.

### Hospital stay

ER resulted in the shortest hospital stay (3.9 d), which aligns with its minimally invasive nature and faster recovery time. LR had the longest hospital stay (9.3 d), likely due to postoperative recovery needs, pain management, and potential for delayed gastric motility following resection. LECS resulted in an intermediate hospital stay (6.7 d), balancing the benefits of minimally invasive techniques with the need for postoperative monitoring. However, the differences in hospital stay across groups were not statistically significant (*P* = 0.877) (**Table 5**).

**Table 5.** Clinical outcomes by surgical approach

**Outcome**	**ER**	**LR**	**LECS**	**Total**	***P***
Success rate	10/11 (90.9%)	11/12 (91.7%)	19/19 (100%)	40/42 (95.2%)	0.438
Complete resection	5/11 (45.5%)	12/12 (100%)	16/19 (84.2%)	33/42 (78.6%)	0.033
Operative time (h)	3.6	2.8	5.1	0.880	
Hospital stay (d)	3.9	9.3	6.7	0.877	
Complications					
Intraoperative perforation	5/11 (45.5%)	0/12 (0%)	0/19 (0%)	5/42 (11.9%)	<0.001
Leakage	0/11 (0%)	0/12 (0%)	0/19 (0%)	0/42 (0%)	
Delayed bleeding	2/11 (18.2%)	0/12 (0%)	0/19 (0%)	2/42 (4.8%)	0.052
SSI	0/11 (0%)	1/12 (8.3%)	1/19 (5.3%)	2/42 (4.8%)	0.656
Other complications	0/11 (0%)	2/12 (12.7%)	1/19 (5.3%)	3/42 (7.1%)	0.289
Hospital stay (d)	3.9	9.3	6.7	0.877	

ER, endoscopic resection; LECS, laparoscopic-endoscopic cooperative surgery; LR, laparoscopic resection; SSI, surgical site infection.

## Discussion

Esophageal and gastric SELs encompass a heterogeneous group of lesions that require careful management to ensure optimal clinical outcomes. Minimally invasive techniques, including ER, LR, and LECS, have been widely adopted to achieve complete tumor removal while preserving gastric function. The present study aimed to compare these three approaches in terms of complete oncological resection, technical success, and postoperative complications among patients treated at King Chulalongkorn Memorial Hospital.

## Efficacy of surgical approaches

Our findings revealed significant differences in complete resection rates among the three techniques. LR demonstrated the highest success rate (100%), followed by LECS (84.2%), while ER had the lowest (45.5%) (*P* = 0.033). These results align with previous studies indicating that laparoscopic surgery provides a more definitive resection, particularly for tumors arising from the muscularis propria layer. The lower complete resection rate in ER suggests that endoscopic techniques may be less effective for deeply located or larger lesions, where achieving clear margins is more challenging and requires higher technical skill [9, 10]. In the present study, the relatively low complete resection rate observed may be attributable to technical limitations. Notably, procedures performed during the early phase of the study demonstrated a lower complete resection rate compared to those conducted later. These findings underscore the importance of procedural proficiency and the learning curve associated with endoscopic techniques, suggesting that operator experience plays a critical role in achieving optimal oncologic outcomes. Determining the layer of involvement by endoscopic ultrasound (EUS) is critical in planning resection techniques [11]. In the present study, all esophageal SELs were managed using ER. Histopathological examination revealed leiomyoma in all cases, with lesion sizes measuring 0.7 cm, 2.5 cm, and 3.0 cm. The smallest lesion was successfully resected using ESD technique, while the 2 larger lesions were resected via the STER technique. Notably, the largest lesion (3.0 cm) demonstrated a positive resection margin, highlighting the potential limitation of ER in achieving complete resection in larger esophageal lesions. However, ER remains an attractive option for smaller, intraluminal tumors due to its lower invasiveness and quicker recovery time [12, 13].

LECS is a hybrid surgical technique that integrates the advantages of endoscopy and laparoscopy, initially introduced by Hiki et al. [14]. Before the development of LECS, LR was the standard approach for treating SELs. However, the external appearance of gastric SELs is often unremarkable, especially in the endophytic pattern, making it challenging to accurately determine resection margins using LR alone [15,16,17]. This limitation may result in either incomplete or excessive resection, potentially increasing the risk of tumor recurrence or leading to postoperative gastric stasis and altered gastric function. The primary advantages of LECS include precise tumor resection with minimal surgical margins, preservation of gastric motility, and maintenance of postoperative quality of life. This is particularly beneficial for SELs located in the EGJ, as it can prevent the need for total or proximal gastrectomy [17]. In the present study, the LECS group comprised classic LECS in 16 out of 19 cases, NEWS in 1 case, and the CLEAN-NET in 2 cases. These newer LECS techniques have been developed to minimize the risk of tumor spillage and reduce the likelihood of peritoneal contamination and subsequent infection or tumor seeding.

## Complications and safety profile

Our results showed a higher delayed bleeding rate in the ER group (18.2%), compared to 0% in LR and LECS. Postoperative bleeding following ER is a recognized complication that can occur shortly after the procedure or up to several weeks postoperatively, with the highest incidence observed within the first 24 h. Clinical manifestations may include hematemesis, melena, hemodynamic instability, and a decline in hemoglobin levels. Several risk factors have been associated with postoperative bleeding, including lesion size, resection defect size, and tumor location. Additionally, patient-related factors, such as advanced age and underlying comorbidities, contribute to an increased risk. Procedural factors, including intraoperative bleeding, adequacy of hemostasis, use of anticoagulant or antiplatelet therapy, and the experience level of the endoscopist, also play a critical role in the likelihood of postoperative hemorrhage. Identifying and mitigating these risk factors is essential to improving patient outcomes and minimizing complications following ER [18, 19]. While endoscopic techniques are considered minimally invasive, they carry a high risk of intraoperative perforation. Intraoperative perforation is a recognized complication of ER with reported incidence rates ranging from 1.2% to 5.2%. Several risk factors have been identified, including lesion size, lesion characteristics, anatomical location, and procedure duration. ER performed for lesions exceeding 20 mm in diameter, deeply infiltrating lesions, or those located in the middle and upper gastrointestinal tract has been associated with a higher risk of perforation. Additionally, the presence of fibrosis at the lesion site, piecemeal resection, and procedures exceeding 2 h have been linked to an increased likelihood of perforation [9, 10, 12, 13, 20]. Careful patient selection, preoperative planning, and advanced endoscopic techniques are essential in minimizing the risk of this complication and ensuring optimal procedural outcomes.

In contrast, in the present study, LECS demonstrated the lowest overall complication rate, reinforcing its safety advantage over ER and LR, which is consistent with prior systematic review and meta-analysis studies [4]. Despite these advantages, LECS has inherent limitations, including the risk of peritoneal contamination with tumor cells or gastric contents due to intentional opening of the gastric wall, as well as the requirement for advanced endoscopic and laparoscopic expertise [21, 22].

## Clinical implications

The findings of the present study highlight the significant role of patient selection in determining the most appropriate surgical approach for the management of esophageal and gastric SELs. Key factors influencing surgical decision-making include tumor growth pattern, anatomical location, depth of tumor, and the technical expertise of the surgeon. For smaller, intraluminal tumors, ER may be a suitable approach; however, preoperative evaluation of tumor depth using EUS is essential to ensure appropriate case selection. Moreover, careful consideration must be given to the risk of perforation, particularly in tumors originating from the muscularis propria layer. Additionally, expertise in endoscopic closure techniques and proficiency in hemostatic management are critical in reducing the risk of intraoperative and postoperative complications.

LR remains a viable and effective approach, particularly for larger or deeply located tumors. However, in the present study, LR was associated with a prolonged hospital stay, consistent with findings from previous meta-analyses [4].

LECS, as an emerging hybrid technique, appears to provide an optimal balance between efficacy and safety, offering precise tumor resection while preserving gastric function and minimizing the risk of postoperative complications.

Several limitations should be acknowledged. First, the sample size was relatively small, which may limit the generalizability of the findings. Second, this retrospective study includes selection bias, as the choice of surgical technique was influenced by surgeon preference based on patient clinical factors, potentially impacting the comparability of outcomes across different approaches. Future research should focus on multi-center studies with larger sample sizes and longer follow-up durations to better understand the long-term benefits and risks associated with each technique.

## Conclusion

In summary, the present study provides a comparative analysis of ER, LR, and LECS in the management of esophageal and gastric SELs. While all three techniques demonstrated a high technical success rate, ER was associated with the lowest rate of complete resection and the highest risk of delayed bleeding. These findings highlight the importance of individualized surgical planning, considering factors such as tumor size, location, and depth of invasion, to optimize clinical outcomes in patients with esophageal and gastric SELs.

## References

[1] Hedenbro JL, Ekelund M, Wetterberg P (1991). Endoscopic diagnosis of submucosal gastric lesions: the results after routine endoscopy. Surg Endosc.

[2] Papanikolaou IS, Triantafyllou K, Kourikou A, Rösch T (2011). Endoscopic ultrasonography for gastric submucosal lesions. World J Gastrointest Endosc.

[3] Aghdassi A, Schröder S, Becker K, Riecken EO, Wiedenmann B (2018). Gastrointestinal stromal tumors: clinical symptoms, location, metastasis formation, and associated malignancies in a single center retrospective study. Dig Dis.

[4] Zhu H, Zhao S, Jiao R, Zhou J, Zhang C, Miao L (2020). Comparison of endoscopic versus laparoscopic resection for gastric gastrointestinal stromal tumors: a preliminary meta-analysis. J Gastroenterol Hepatol.

[5] Meier B, Schmidt A, Glaser N, Meining A, Walter B, Wannhoff A (2020). Endoscopic full-thickness resection of gastric subepithelial tumors with the gFTRD-system: a prospective pilot study (RESET trial). Surg Endosc.

[6] Guo JT, Zhang JJ, Wu YF, Liao Y, Wang YD, Zhang BZ (2021). Endoscopic full-thickness resection using an over-the-scope device: a prospective study. World J Gastroenterol.

[7] de Brito SO, Libânio D, Pinto CMM, de Araújo Teixeira JPPO, de Araújo Teixeira JPM (2022). Efficacy and safety of laparoscopic endoscopic cooperative surgery in upper gastrointestinal lesions: a systematic review and meta-analysis. GE Port J Gastroenterol.

[8] Teng TZJ, Ishraq F, Chay AFT, Tay KV (2023). Lap-Endo cooperative surgery (LECS) in gastric GIST: updates and future advances. Surg Endosc.

[9] Bang CS, Baik GH, Shin IS, Suk KT, Yoon JH, Kim DJ (2016). Endoscopic submucosal dissection of gastric subepithelial tumors: a systematic review and meta-analysis. Korean J Intern Med.

[10] Chen Q, Yu M, Lei Y, Zhong C, Liu Z, Zhou X (2020). Efficacy and safety of endoscopic submucosal dissection for large gastric stromal tumors. Clin Res Hepatol Gastroenterol.

[11] Sharzehi K, Sethi A, Savides T (2022). AGA clinical practice update on management of subepithelial lesions encountered during routine endoscopy: expert review. Clin Gastroenterol Hepatol.

[12] Goto O, Higuchi K, Koizumi E, Iwakiri K (2025). Advancements in endoscopic treatment for gastric subepithelial tumors. Gut Liver.

[13] Li DM, Ren LL, Jiang YP (2020). Long-term outcomes of endoscopic resection for gastric subepithelial tumors. Surg Laparosc Endosc Percutan Tech.

[14] Hiki N, Yamamoto Y, Fukunaga T, Yamaguchi T, Nunobe S, Tokunaga M (2008). Laparoscopic and endoscopic cooperative surgery for gastrointestinal stromal tumor dissection. Surg Endosc.

[15] Namikawa T, Hanazaki K (2015). Laparoscopic endoscopic cooperative surgery as a minimally invasive treatment for gastric submucosal tumor. World J Gastrointest Endosc.

[16] Matsuda T, Nunobe S, Ohashi M, Hiki N (2017). Laparoscopic endoscopic cooperative surgery (LECS) for the upper gastrointestinal tract. Transl Gastroenterol Hepatol.

[17] Hiki N, Nunobe S (2019). Laparoscopic endoscopic cooperative surgery (LECS) for the gastrointestinal tract: updated indications. Ann Gastroenterol Surg.

[18] Yang CH, Qiu Y, Li X, Shi RH (2020). Bleeding after endoscopic submucosal dissection of gastric lesions. J Dig Dis.

[19] Guo Z, Miao L, Chen L, Hao H, Xin Y (2018). Efficacy of second-look endoscopy in preventing delayed bleeding after endoscopic submucosal dissection of early gastric cancer. Exp Ther Med.

[20] Oda I, Suzuki H, Nonaka S, Yoshinaga S (2013). Complications of gastric endoscopic submucosal dissection. Dig Endosc.

[21] Goto O, Takeuchi H, Kitagawa Y, Yahagi N (2016). Endoscopic submucosal dissection (ESD) and related techniques as precursors of “New Notes” resection methods for gastric neoplasms. Gastrointest Endosc Clin N Am.

[22] Tsai TC, Meireles OR (2019). Combined surgical and endoscopic approaches to full-thickness resection. Tech Gastrointest Endosc.

